# Reference genomes for BALB/c Nude and NOD/SCID mouse models

**DOI:** 10.1093/g3journal/jkad188

**Published:** 2023-08-18

**Authors:** Emanuel Schmid-Siegert, Mengting Qin, Huan Tian, Bulak Arpat, Bonnie Chen, Ioannis Xenarios

**Affiliations:** JSR Life Sciences, NGS-AI CH Division Route de la Corniche 3, 1066 Epalinges, Switzerland; JSR Life Sciences, NGS-AI CN Division, Industrial Park, Suzhou, Jiangsu 215000, P.R. China; JSR Life Sciences, NGS-AI CN Division, Industrial Park, Suzhou, Jiangsu 215000, P.R. China; JSR Life Sciences, NGS-AI CH Division Route de la Corniche 3, 1066 Epalinges, Switzerland; JSR Life Sciences, NGS-AI CN Division, Industrial Park, Suzhou, Jiangsu 215000, P.R. China; JSR Life Sciences, NGS-AI CH Division Route de la Corniche 3, 1066 Epalinges, Switzerland

**Keywords:** *Mus musculus*, BALB/c Nude, NOD/SCID

## Abstract

Mouse xenograft models play a vital role in tumor studies for research as well as for screening of drugs for the pharmaceutical industry. In particular, models with compromised immunity are favorable to increase the success of transplantation, such as, e.g. NOD/SCID and BALB/c Nude strains. The genomic sequence and alterations of many of these models still remain elusive and might hamper a model’s further optimization or proper adapted usage. This can be in respect to treatments (e.g. NOD/SCID sensitivity to radiation), experiments or analysis of derived sequencing data of such models. Here we present the genome assemblies for the NOD/SCID and BALB/c Nude strains to overcome this short-coming for the future and improve our understanding of these models in the process. We highlight as well first insights into observed genomic differences for these models compared to the C57BL/6 reference genome. Genome assemblies for both are close to full-chromosome representations and provided with liftover annotations from the GRCm39 reference genome.

## Introduction

Reference genomes have been essential in many research fields and with the advances in high throughput sequencing, detailed characterizations of genomes become more readily available (Miga *et al.* 2020), including the mouse genome (Gregory *et al.* 2002; Ferraj *et al.* 2022). One field where understanding the biology, and therefore genomic makeup, of the model strain remains key, is in the generation of immunocompromised mice. These mouse strains play a crucial role in establishing mouse tumor models such as cell line-derived xenografts (CDXs), patient-derived xenografts (PDXs) and homografts in syngeneic mice (Li *et al.* 2017; Olson *et al.* 2018; Okada *et al.* 2019). The genetic background of a strain profoundly affects the phenotype and potential applications of a mouse model, which contributes to defects in innate immunity such as reduced natural killer (NK) cell activity, absence of circulating complement, and deficits in macrophages as well as antigen-presenting cells (Chuprin *et al.* 2023).

The combination of such defects are often obtained through crosses of single defect models such as in the case of NOD and SCID crossed to a NOD/SCID mouse model. Some of the defects are genomically well defined and derive for example from a single-point mutation, others from transgenes or less well characterized genomic alterations. Subsequently, the genomic sequence and alterations of some of these models are more elusive and might hamper its further genetic and immunonolgic optimization. Based on PDX-recycled data, we present here the genomic characterization of the BALB/c Nude and NOD/SCID models to facilitate their understanding and usage. The nonobese diabetic severe combined immunodeficiency (NOD/SCID, Bosma *et al.* 1983)-based immunocompromised mice lack both, functional T- and B-lymphocytes and present a dysfunctional NK cell activity. BALB/c Nude mice on the other hand have lower macrophage-mediated phagocytosis of human cells and a deficiency of mature T-lymphocytes, which makes them together with the nude phenotype suitable for transplantation.

Sequencing data from multiple PDXs was pooled and the mouse strain assembled after eliminating human sequences. As tissue derived from PDX samples is always a mixture of host and graft material, this is reflected similarly in the obtained sequencing data. Depending on multiple factors (location, type of tumor, experimentator, and more), this ratio has been seen to be as low as <1% but as well high as beyond 60% (Woo *et al.* 2019; Karamboulas *et al.* 2020). The latter is less desired for sequencing as it lowers the targeted assay coverage and complicates analysis of the data. Recent progress has been made to evaluate this already prior sequencing by using a high-throughput, low-cost, NGS-based screening approach (Chen *et al.* 2020). This approach has not been applied to the models used in this study.

We present a strategy to make use of the unwanted host-derived data to reconstruct its corresponding genome de novo and present close to full-chromosome assemblies for the BALB/c Nude and NOD/SCID strains as a resource to improve our understanding of their genomic makeup and facilitate their use in xenograft-related studies.

## Materials and methods

### PacBio HIFI sequencing

High-molecular-weight gDNA was prepared from PDX tissue. The cells were lysed using Tissue Lyser II (Quiagen, Hilden, Germany) and the DNeasy Blood & Tissue Kit 250 (Quiagen). The resulting gDNA was verified to be >15 μg and >10 kbp long. DNA samples were purified and concentrated using AMPure PB beads (PacBio, Menlo Park, USA) to remove small fragments and impurities prior to library preparation. Library construction for HIFI protocol was done using SMRTbell Express Template Prep Kit 2.0 (PacBio), followed by another round of AMPure PB Beads Purification and Bluepippin size selection (<8 kbp). Samples were sequenced with Sequel II Sequencing Kit 2.0 on PacBio Sequel II (PacBio).

### Bionano sample and data preparation

DNA was extracted from tissue using the Bionano Prep SP Tissue and Tumor DNA Isolation Kit (Bionano, San Diego, USA) and a Tissue Ruptor (Quiagen) targeting a final concentration of 50–120 ng/μL. DLE-1 labeling was done using the Bionano Prep DLS Labeling Kit with final concentration targeted at 4–12 ng/μL. Data collection was performed using Saphyr 2nd generation instruments.

### PacBio HIFI analysis

Consensus sequences were generated for all reads using ccs (v6.0.0; -j 0 –all –hifi-kinetics) and split into HIFI and low-quality reads using bamtools (Barnett *et al.* 2011; v2.5.1; filter -tag “rq:<0.99”). uBAM entries were translated into fastq format using bam2fastq (v) and classified using xengsort (Zentgraf and Rahmann 2021; v0.9.1; index generated using mouse genome and transcriptome as well as human genome and transcriptome: -p 4 –fill 0.94 -k 25 -P 4800000000 3FCVbb:u –hashfunctions linear945:linear9123641:linear349341847).

### Genotyping and single nucleotide variant analysis

De novo variant calling was done using deepvariant (Poplin *et al.* 2018; v1.1.0; model_type=PACBIO) using the HIFI host reads aligned to GRCm39 with pbmm2 (v1.4.0; –preset CCS). For the pileup-based analysis, we used the tool abc (v0.2.1) providing the 2 marker mutation positions and the same alignments. For single nucleotide variant (SNV) analysis, regions with low complexity were removed using dust (v0.1; -w 64) and tandem repeat finder (v4.09; trf 2 5 7 80 10 50 2000 -f -d –m; followed with TRFdat_to_bed.py) and bedtools (Quinlan and Hall 2010; v2.29.0).

### Structural variant analysis

Analysis was based on above-described alignments using pbsv (v2.4.0). Regions for which mapping quality was ≤5 for more than 2 reads within a range of 10 bp were removed as well as structural variants (SVs) smaller than 50 bp, or >5% allelic frequency or less than 3 reads supporting it (SURVIVOR; v1.0.7). Events in-between samples were compared using SURVIVOR merge within 1,000 bp range, same type and same direction of the event.

### Genome assembly

The HIFI reads were assembled using hifiasm (Cheng *et al.* 2021; yv0.12-r304) and were aligned against GRCm39 using mashmap (Ondov *et al.* 2019; v2.0, -k 16 –s 1000 -f one-to-one –perc_identity 85). Contigs for which no alignment ≥5 kbp was found were extracted (427 contigs in NOD/SCID mice and 1,542 in BALB/c Nude) and screened again using xengsort to check if assembly generated contigs which now clearly identified as human derived. Contigs which were not clearly host assigned were removed. The remaining ones were screened using mash screen (v2.0, screen -w -p 30) against the refseq collection (RefSeq88n). It suggested there was a presence of endogenous mouse viruses in some, together with other murine viruses—but no graft similarities were anymore observed. These contigs were added back to the assemblies. We then used one round of haplotig purging as describe in purge_dups (v1.0.1).

### Bionano scaffolding

For the BALB/c Nude genotype, the sample11 provided enough material to generate a matching Bionano run. For the NOD/SCID genotype, this was not the case and we screened further 15 PDX for which lllumina WGS had been conducted. We identify a matching sample, Sample12, and run Bionano on it. We combined the genome sequence of the human and mouse genome and added in silico DLE-1 sites using their “fa2cmap_multi_color.pl” tool (Solve3.6.1_11162020). After de-noising, we aligned the molecules against this combined reference and only kept molecules aligning preferentially to the mouse genome reference. These molecules were then assembled using Bionano’s tool “pipelineCL.py” (Solve3.6.1_11162020) and used to scaffold the matching, cleaned genome assembly. Assemblies have been screened with the NCBI Foreign Contamination Screen (Astashyn *et al.* 2023; v0.4.0-3-g8096f62 ; –tax-id 10090) and made available under the BioProject PRJNA944543.

### Genome annotation

GRCm39 annotation (Mus_musculus.GRCm39.104.gtf_db) was lifted over using liftoff (v 1.5.1, -copies) for both strain genome assemblies.

### Genome assessment

BUSCO (Manni *et al.* 2021a; v5.2.2) based on Glires dataset (containing 13,798 Busco’s group on 24 genomes) was used to evaluate the completeness of each strain genome assembly. For the BUSCO transcriptome analysis, liftover annotation were used together with gffread to extract the transcript FASTA entries (v0.12.7, feature “transcripts”). BUSCO transcript was run on these transcript catalogs (BALB/c Nude: 140–147 transcripts, NOD/SCID: 139–795 transcripts, GRCm39: 142–434 transcripts).

## Results and discussion

### Sample characterization

Due to their length combined with high quality, we chose PacBio HIFI reads for the genome assembly process. This feature combination reduces significantly the possibility to accidentally introduce human sequences into the assembly step compared to short reads or more error-prone long reads. The HIFI reads were screened with xengsort, a fast and accurate xenograft sorting algorithm (Zentgraf and Rahmann 2021), and reads which were unambiguously identified as graft (human) were removed. All the other reads were kept for further analysis, which included as well the categories “both” and “ambiguous.” The former including reads which carry unique kmers for both organism, whereas the latter containing kmers which are equally present in both organism—e.g. telomeric-, centromeric-, or very conserved regions. This resulted in a 19–62% observed mouse-content per sample (Supplementary Table 1) in agreement with previous reports (Rossello *et al.* 2013; Chen *et al.* 2021). Median mouse-content were consistent across BALB/c Nude (24.5%) and NOD/SCID (31%) samples.

We confirmed the genotype of each PDX sample based on known marker mutations. The BALB/c Nude mouse model is characterized by a 1 bp deletion in the exon 3 of the Foxn1 gene which leads to a truncated protein and subsequently the nude phenotype (Schlake 2001). The NOD/SCID mice should not carry the nude mutation but another marker mutation which is in the 2nd to last exon of the Prkdc gene and results in an early stop codon of that gene (Blunt *et al.* 1996). This NOD/SCID mutation is not present in the BALB/c Nude model. We systematically analyzed these 2 positions for all 13 samples using 2 approaches based on the mapping of HIFI reads to the reference GRCm39 genome: (1) de novo SNV calling on the entire genome and (2) pileup analysis at the marker positions without any genotype inference. The expected genotype was confirmed in all samples by both approaches.

Prior to pooling samples for the assembly process, we investigated further their similarity and variation for both, within- and in-between-strains. To this end, we called SNVs and SVs from the alignment of the HIFI reads against the GRCm39 reference genome using deepvariant (Poplin *et al.* 2018) and PacBio’s pbsv algorithm (Wenger *et al.* 2019), respectively. Variants in tandem repeated regions in the reference genome were removed, as well as in regions of problematic alignments. To further reduce noise due to shallow depth, variants with low confidence scores were excluded and only homozygous germline variants were further analyzed. Comparison of number of shared events (stranded) between samples identified a clear separation between BALB/c Nude and NOD/SCID mice using SVs (Supplementary Fig. 1) and SNVs (Supplementary Fig. 2). Within strain, samples were very similar to each other, underlining the low sample heterogenity on the genomic makeup. Interestingly, less variant of each type were overall detected in BALB/c Nude mice compared to NOD/SCID mice.

### Global variant characterization

**Fig. 1.**
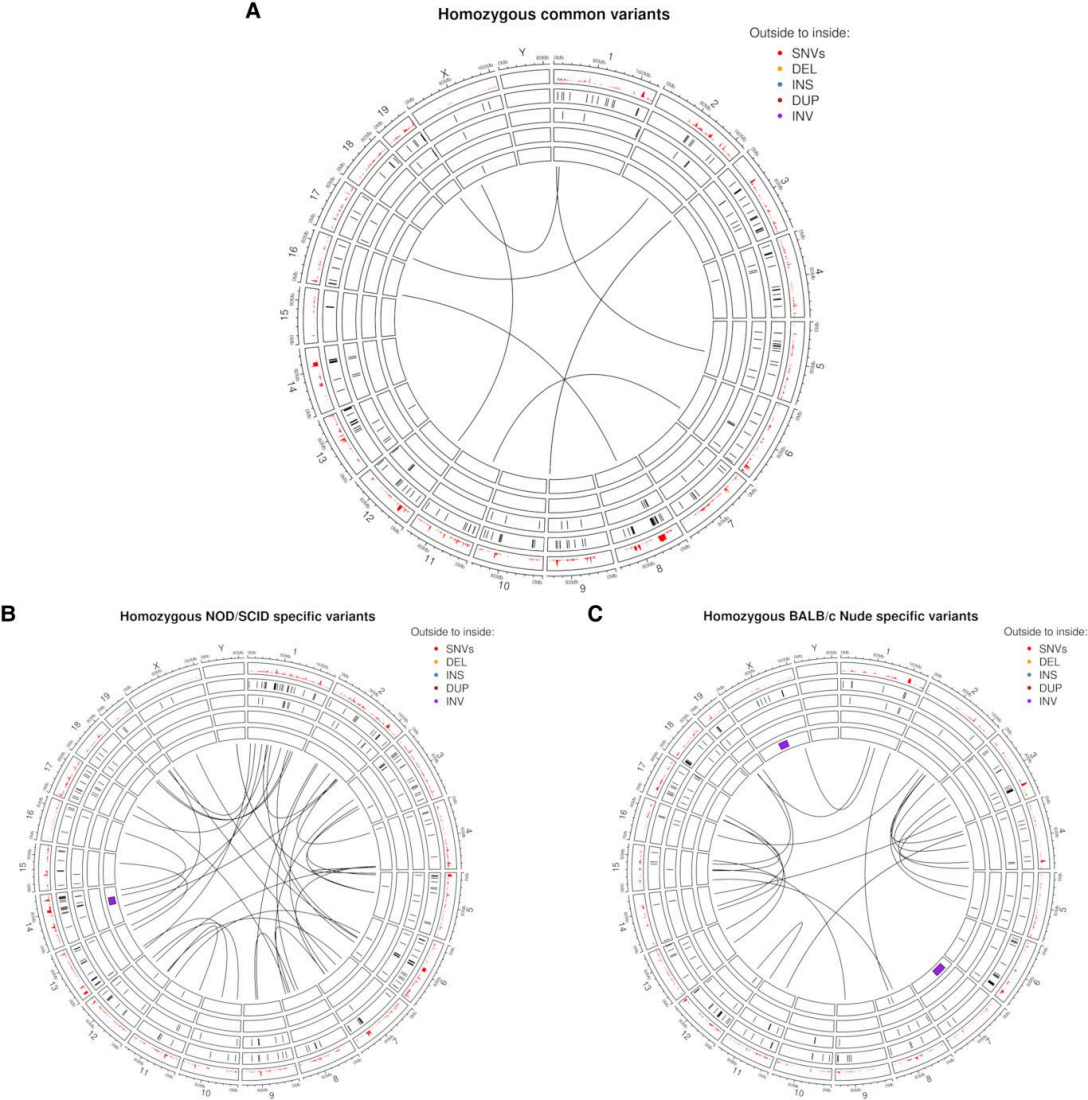
Systematic comparison of homozygous variants in BALB/c Nude and NOD/SCID mice. Homozygous SNVs and SVs were identified relative to the reference genome GRCm39 and were visualized in circular plots. a) Common variants in BALB/c Nude and NOD/SCID mice. Variants specific for NOD/SCID and BALB/c Nude strains were plotted in b) and c), respectively. SNV density is reported in 1 Mbp windows along the GRCm39 reference genome. Chromosomes are organized into sectors indicated by their label, start and end positions at the outside. Color codes indicate different types of variants (DEL, deletion; INS, insertion; DUP, duplication; INV, inversion in that order from the outer to the inner circle). Translocations are plotted in the middle of the circular plots as junctions between the breakpoints.

To further analyze and understand the genomic variations, we grouped homozygous SV- and SNV-events into 3 groups: events observed (1) commonly in both strains, (2) uniquely in BALB/c Nude, and (3) uniquely in NOD/SCID (Fig. 1). As expected, no events were reported on the Y chromosome which is absent in our uniquely female sample collection. Similar to SNVs, SV events were more present in the NOD/SCID mice compared to the BALB/c Nude ones, most prominent for deletion and translocation events.

Few clusters can be noticed where a combination of apparently frequent events is occurring. For example, on chromosome 8 around 20 Mbp an accumulation of SNVs and SVs for both genotypes is noticeable (Fig. 1a). One plausible reason for such accumulation of SNV calls in this region could be due to less accurate representation of the genomic architecture as supported by the nearby SV events. SNVs which are called in such regions might often be flawed through misrepresentation of the genomic structure and advocate for the need of strain-specific genome assemblies. Only six translocations between chromosomes were detected to be common for both mouse strains in reference to the GRCm39 genome assembly (Fig. 1a).

Analyzing the NOD/SCID specific variants (Fig. 1b), we found a cluster of SNVs and SV on chromosome 7 around 100 Mbp. Interestingly, this region overlaps with the cluster of Olfr members (olfactory genes) which are known to be highly diversified in mice (Young 2002). Another example is a hotspot of genomics rearrangements, containing a combination of inversion, deletion, and insertion events, that manifests itself on chromosome 14 around 80 Mbp. Such combination of different SV events often stem from the inability of the SV caller to resolve complex rearrangements and are better accessible through a de novo assembled genome instead.

### Strain-specific genome assemblies

Based on the results of the genotyping, SV and SNV analysis, we pooled data from matching samples for each strain (Supplementary Table 1) and assembled the BALB/c Nude and NOD/SCID genomes. The coverage of nonhuman reads was equivalent to 44.5-fold and 47.2-fold the mouse genome size, respectively.

Due to the PDX nature of the samples, it was important to verify the absence of human sequences pre- but as well post-assembly. The raw data were xengsorted and we aligned assembled contigs against the GRCm39 reference genome, screening nonmapping contigs further with the xengsort algorithm. Only contigs that could be clearly assigned as “host” genome-derived were kept. This subset was further screened against the RefSeq collection release 88 (O’Leary *et al.* 2016) using Mash Screen (Ondov *et al.* 2019), which did not identify any major contaminants such as, e.g. bacterial genomes.

To further improve the continuity of the assembled HIFI contigs, we used Bionano optical maps as anchors for scaffolding. For each strain, new genomic DNA was prepared from a new PDX sample after confirming its genotype as described earlier. One Bionano flow-cell was used for each strain and raw molecules were competitively mapped against human and mouse in silico maps. Molecules with higher similarity to the host were then assembled and used for the scaffolding.

Bionano molecules of the NOD/SCID individual showed 15% mouse-content and provided in total 185 Gbp of filtered molecules with a molecule N50 of 335 kbp. These molecules were assembled into 118 maps spanning 2.6 Gbp with a N50 of 74 Mbp. For the BALB/c Nude, individual 75% of molecules were of mouse origin and they summed up in 770 Gbp filtered molecules with a N50 of 305 Kbp, which were assembled into 60 maps of 2.586 Gbp and a map N50 of 96.8 Mbp. These maps were then used to scaffold the HIFI assemblies into chromosome-scale sequences (Table 1). Scaffolded assemblies were screened with the NCBI Foreign Contamination Screen (Astashyn *et al.* 2023) and flagged mitochondria genomes, as well as one chimeric BALB/c Nude 15 kbp contig, were removed.

**Table 1.** Basic genome assembly statistics.

Sample	Scaffolds	Unplaced contigs	Max. scaff. (Mbp)	Sum (Gbp)	NG50 (Mbp)
BALB/c Nude	37	1033	167	2.75	167.3
NOD/SCID	28	270	195	2.72	121.8

Statistics describing the genome assemblies for BALB/c Nude and NOD/SCID strains after cleaning and scaffolding with Bionano data.

The Bionano-based scaffolding resolved for both strains multiple chromosomes entirely, except for the telocentric centromeres and telomeres (Supplementary Figs. 3 and 4). The unplaced contigs consist of difficult to resolve regions such as large repeats, mobile elements, and telomere/centromere regions. The possibility that some haplotigs could be attributed to individual sample variation was not further investigated. The number of unplaced contigs was noticeably higher in the BALB/c Nude assembly compared to the NOD/SCID assembly (Table 1). We did not generate gene predictions but concentrated on lifting over known gene features to facilitate the adoption of the assemblies for the community. To that end, we used liftoff (Shumate and Salzberg 2021) to find for each CDS of GRCm39 the boundaries matching on our assemblies and annotate them accordingly in a GTF file (Supplementary Table 2).

The BUSCO near-universal single-copy orthologs (Manni *et al.* 2021b) are often used to assess whether core genes are represented in an assembly and to what extent they are unique or repeated. The BUSCO results for the genome assemblies of both immunodeficient strains, BALB/c Nude and NOD/SCID, were on par with the results for the C57BL/6 reference (GRCm39) using either the genome assemblies (Table 2) or liftover-derived transcriptomes (Supplementary Table 3). BALB/c Nude had a slightly higher number of duplicated elements which might be contained within haplotigs that were not entirely purged.

**Table 2.** BUSCO scores for genome assemblies.

	BALB/c Nude (%)	NOD/SCID (%)	GRCm39 (%)
Total complete	96.4	96.5	96.5
Complete single copy	92.3	93.6	93.6
Complete duplicated	4.1	2.9	2.9
Fragmented	0.7	0.6	0.6
Missing	2.9	2.9	2.9

BUSCO assessment of BALB/c Nude, NOD/SCID, and C57BL/6 reference genome assemblies (GRCm39). Assemblies were analyzed against the glires_odb10 collection which contains 13–798 groups in total.

### Discordant regions between mouse strains

The gene annotation liftover approach provides an estimate of the genes that are absent in our assemblies given the GRCm39 annotation. In total, 981 and 1,157 genes were missing for BALB/c Nude and NOD/SCID, respectively, after lifting over non-Y chromosome annotations from the reference genome. Out of these, 737 were common for both strains, most of which represented pseudo-genes and long noncoding RNAs (see GitHub:“common_missingLiftover.bed”) as well as 89 protein-coding genes. Out of the missing protein-coding transcripts, many are “Gm” prefixed gene names, indicating that they are potentially active transcripts which have no proper name for the moment. Excluding the Y chromosome genes, 98.1 and 97.9% of GRCm39 annotated genes could be identified in BALB/c Nude and NOD/SCID assemblies, respectively.

**Fig. 2.**
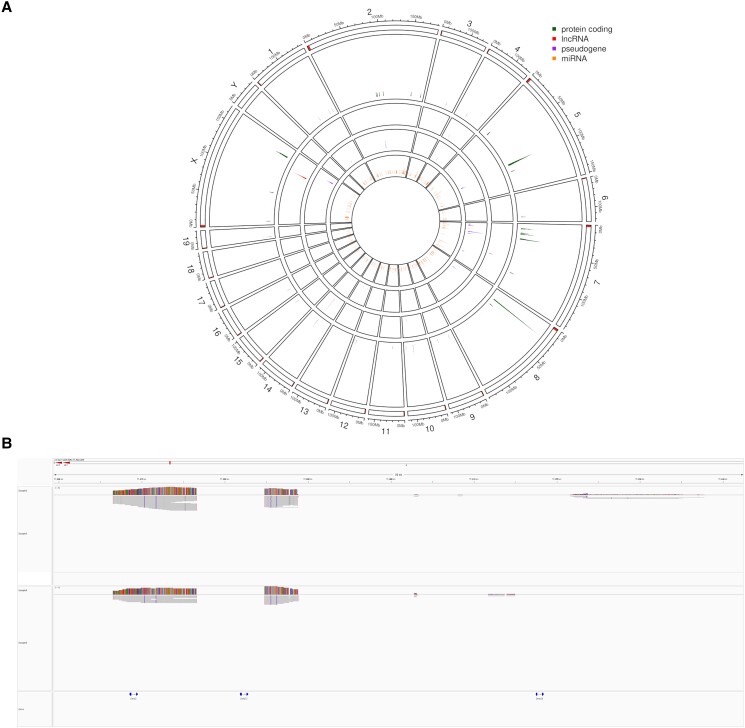
Identification of reference features that are absent in BALB/c Nude and NOD/SCID mice. a) Circular visualization of annotated reference features that could not be identified in the genome assemblies of BALB/c Nude and NOD/SCID strains. The organization of chromosomes is similar to that in Fig. 1, except that the slices representing the 5 chromosomes with most number of missing protein-coding features were enlarged for clarity. Color codes indicate types of features. b) Close-up view of the highlighted region containing members of the defensin alpha cluster on chromosome 8 (21,859,396-21,942,429; GRCm39). HIFI reads of one representative NOD/SCID (sample 3, upper panel) and BALB/c Nude (sample 6, lower panel) individual aligned to GRCm39 genome using minimap2 and visualized in IGV (Integrative Genomics Viewer) browser in upper and lower panels, respectively. Mismatch coloring was deactivated for clarity.

Looking at the distribution of commonly missing genes along the chromosomes, one can identify clusters (Fig. 2a). In regard to missing protein-coding genes, chromosomes 7, 8, and 5 rank highest with 15, 14, and 12 common missing features, respectively. Chromosome 7 is missing 6 members of the Scgb family (secretoglobin, family 1B), 3 Zscan members (Zinc finger and SCAN domain containing protein 4), 5 Vmn1r (vomeronasal 1 receptor) members, and Gvin1 (GTPase, very large interferon inducible). Chromosome 8 misses for both strains almost exclusively Defa (defensin, alpha) members (Fig. 2b) and Gm10131 (predicted pseudogene 10131). Finally, chromosome 5 is more heterogenous with 5 Pramel members (PRAME like), 4 Gm* and Vmn2r15 (vomeronasal 2, receptor 15), Cacna2d1 (Calcium Voltage-Gated Channel Auxiliary Subunit Alpha2delta 1), and AA792892 (expressed sequence AA792892).

**Fig. 3.**
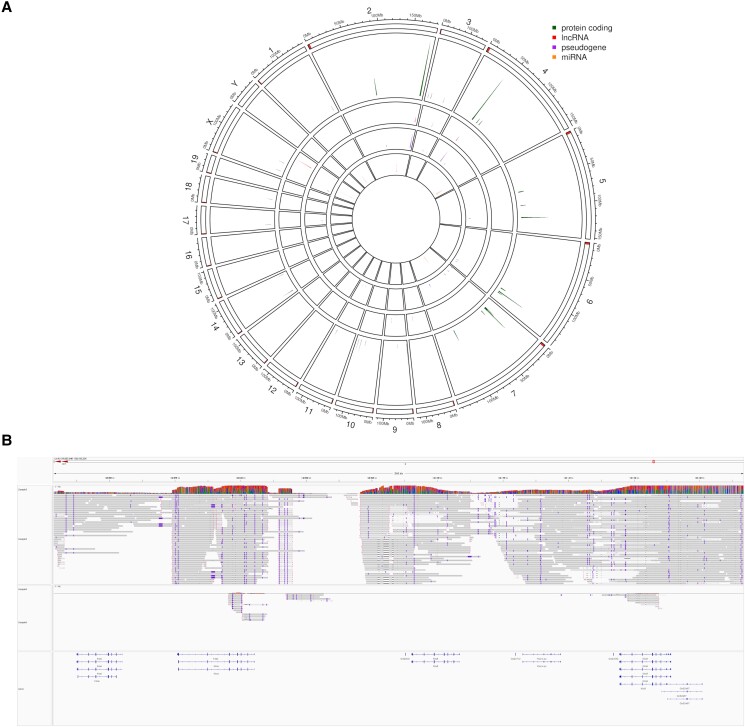
Identification of reference features that are specifically absent in BALB/c Nude strain. a) GRCm39 features that are absent specifically in BALB/c Nude genome assembly were plotted similarly to that in Fig. 2. b) Close-up view of Klra genes region on chromosome 6 (129,982,946-130,193,356; GRCm39). HIFI reads of one representative NOD/SCID (sample 3, upper panel) and BALB/c Nude (sample 6, lower panel) individual were aligned to GRCm39 genome using minimap2 and visualized similarly to that in Fig. 2.

Looking at strain-specific missing coding genes, we found a BALB/c Nude specific absent Klra (known as well as Ly49) cluster on chromosome 6 around 130 Mbp (Fig. 3a) which includes Klra10, Klra4, Klra6, Klra8, and Klra9. These genes are present in the NOD/SCID strain, but Klra homology compared to GRCm39 seems low, as indicated by many mismatches, and clipped long reads (Fig. 3b). Composition of natural killer gene complex genes and in particular Klar genes has been described to vary for different strains, with BALB/c Nude showing the most prominent differences and absence of Klra genes (Higuchi *et al.* 2010), which we can observe here as well.

## Conclusion

Here we assembled and annotated genomes for the NOD/SCID and BALB/c Nude strains, characterizing most of the chromosomes to a chromosome-level scale. The vast majority of chromosomes are colinear with the existent C57BL/6 strain GRCm39 genome assembly. Both, raw-data SV analysis as well as genome comparison, identified though regions with large structural alterations, including olfactory genes and a BALB/c Nude specifically absent Klar gene cluster. The low homology Klar gene cluster in NOD/SCID, but especially the complete absence of multiple Klar genes of the NK cluster in BALB/c nude provide insights into the genomic alterations potentially explaining the previously observed reduced NK activity in this strain. The link between residual NK activity and reduced xentransplantation success remains the motivation of continuous constant effort to reduce NK activity further (Panaampon *et al.* 2021). The availability of a genomic de novo assembly and thereby a genomic map provides the tools for a more profound studies of that immunological phenotype.

## Supplementary Material

jkad188_Supplementary_Data

## Data Availability

The genome assemblies are available under the BioProject PRJNA944543, with the BALB/c Nude sample SAMN33748820 and the NOD/SCID sample SAMN33781945. PacBio HIFI consensus sequences of the mouse part have been deposited under the same BioSamples at SRA. Gene liftover annotation tracks (GTF) and missing liftover genes (TXT) can be found on github. Supplementary material is available at G3 online.
