# Supplementary material for: Reference genomes for BALB/c Nude and NOD/SCID mouse models
Source: G3 (Bethesda). 2023 Aug 18;13(10):jkad188. doi: 10.1093/g3journal/jkad188 (PMC10542179; doi:10.1093/g3journal/jkad188)
Supplement: jkad188_Supplementary_Data [file jkad188_supplementary_data.zip › Supplemental_Material_G3-2023-404267.pdf]

1 Supplementary Material

sample	Mouse-content	Genotype
Sample1	25%	NOD/SCID
Sample2	22%	NOD/SCID
Sample3	62%	NOD/SCID
Sample4	32%	NOD/SCID
Sample5	31%	NOD/SCID
Sample6	33%	BALB/c Nude
Sample7	20%	BALB/c Nude
Sample8	26%	BALB/c Nude
Sample9	28%	BALB/c Nude
Sample10	19%	BALB/c Nude
Sample11	23%	BALB/c Nude

Table S1: **Characterization and genotyping of samples for genome assembly**. Table of samples chosen for the genome assembly of BALB/c Nude and NOD/SCID genotypes. For each sample the mouse content and it's genotype was analyzed based on WGS HIFI data. Genotype specific mutations in FOXn1 and prkdc genes were used for the genotyping.

strain	BALB/c Nude	NOD/SCID
total genes	53847	53847
missing Genes	981	1157
lift-over genes	52866	52690
genes <90 %	462	546
genes >=90% & <95%	201	237
genes >=95% & <99%	908	1023
genes >=99%	51295	50884

Table S2: **Liftover success of GRCm39 annotated gene-features for genome assembly of each strain**. Liftover calculates gene coverage as the sum of all it's transcripts covering the gene. From a total of 55416 genes in GRCm39, 1569 were removed prior lift-over as they were located on the Y-chromosome.

	BALB/c Nude assembly	NOD/SCID assembly	GRCm39
Total Complete	99.7 %	99.7 %	99.9 %
Complete single copy	39.8 %	39.8 %	39.9 %
Complete duplicated	59.9 %	59.9 %	60 %
Fragmented	0.1 %	0 %	0.0 %
Missing	0.2 %	0.3 %	0.1 %

Table S3: **BUSCO scores for annotation derived transcriptomes** . BUSCO assessment of BALB/c Nude, NOD/SCID and C57BL/6 (GRCm39) reference transcriptomes. Transcriptome for BALB/c Nude and NOD/SCID were derived from lift-over annotation and all 3 were compared using "glires_odb10" collection which contains 13'798 groups in total.

Figure S1: **Structural variation events shared between samples**. Heatmap comparing >1kbp homozygous SVs called individually for each sample using HIFI reads mapped against the GRCm39 genome assembly. Plotted are number of shared SVs between each sample. SVs are of same type, same direction and within a 1kbp maximal distance to each other.

Figure S2: **Single nucleotide variants shared between samples**. Heatmap comparing homozygous SNVs between each sample using HIFI reads mapped against the GRCm39 genome assembly. Plotted are the number of shared SNVs between samples.

Figure S3: **Coverage analysis of NOD/SCID genome assembly**. Comparison of large segmental alignments between NOD/SCID genome assembly and GRCm39 reference assembly. Bold rectangles represent the GRCm39 chromosome space and color filling the matched assembly regions. Minimap2 alignment was done with "-x asm5" , alignments filtered for query and target contigs lengths $> 1e7$ and minimal match length $> 1e4$. Reference genome covered regions were visualized using pafr (version "115d2b1be6cdab5679a81a536200a03cef1ba0e9").

Figure S4: **Coverage analysis of balb-c genome assembly**. Comparison of large segmental alignments between balb-c genome assembly and GRCm39 reference assembly. Bold rectangles represent the GRCm39 chromosome space and color filling the matching assembly regions. Minimap2 alignment was done with "-x asm5" , alignments filtered for query and target contigs lengths $>$ $1e7$ and minimal match length $> 1e4$. Reference genome covered regions were visualized using pafr (version "115d2b1be6cdab5679a81a536200a03cef1ba0e9").
