# Supplementary figures and images for: Reference genomes for BALB/c Nude and NOD/SCID mouse models
Source: G3 (Bethesda). 2023 Aug 18;13(10):jkad188. doi: 10.1093/g3journal/jkad188 (PMC10542179; doi:10.1093/g3journal/jkad188)

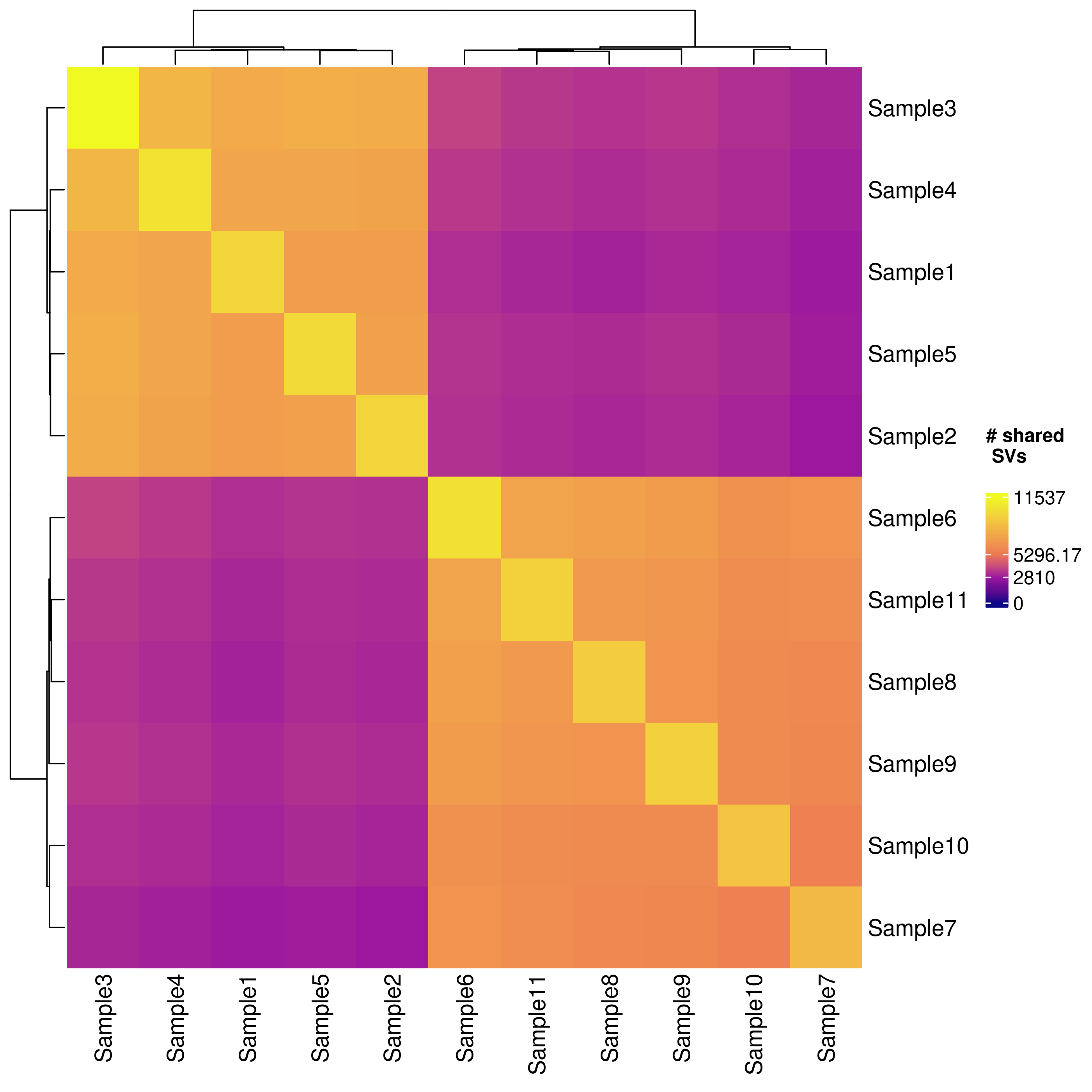

Supplement: jkad188_Supplementary_Data [file jkad188_supplementary_data.zip › Figure_S1_G3-2023-404267.png]

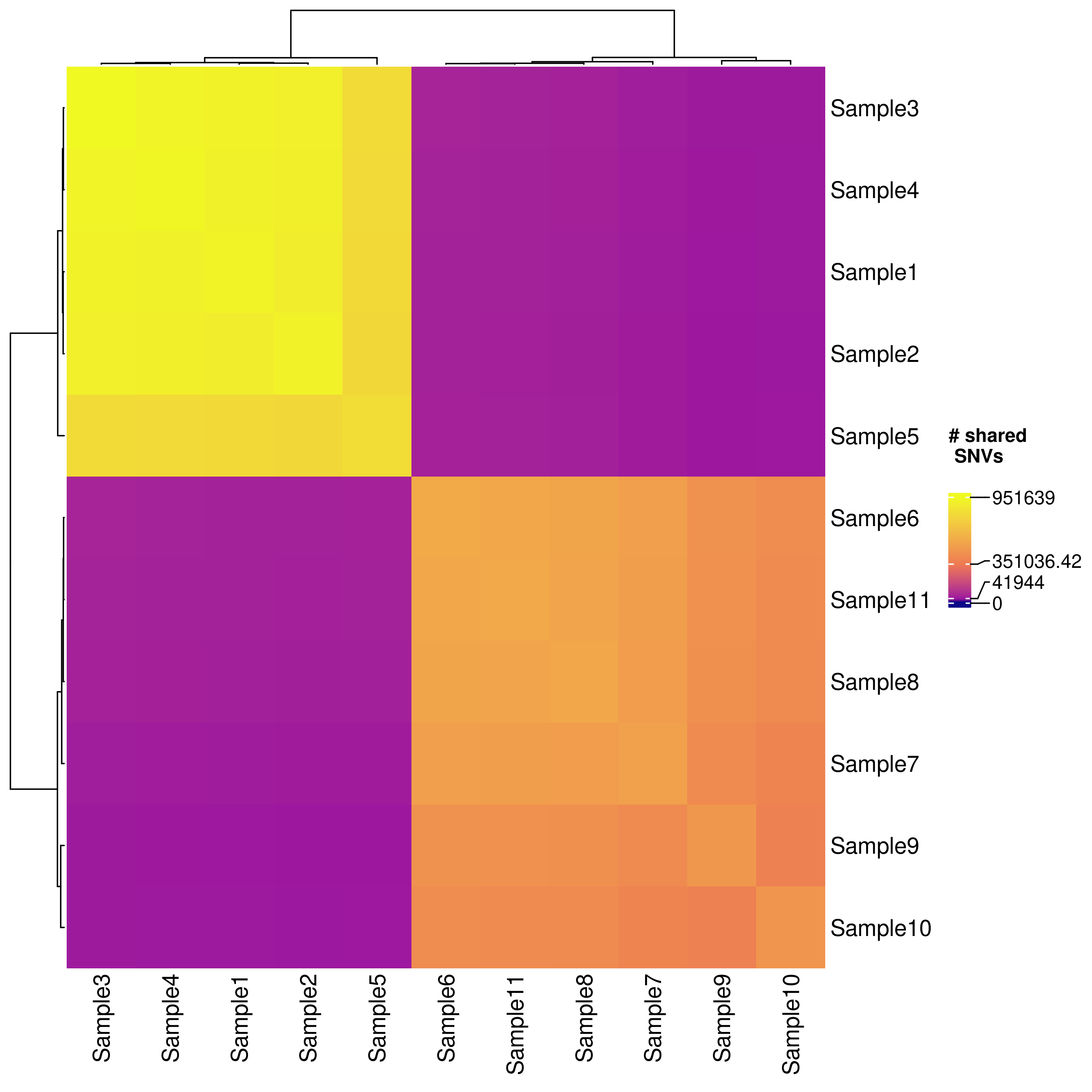

Supplement: jkad188_Supplementary_Data [file jkad188_supplementary_data.zip › Figure_S2_G3-2023-404267.png]

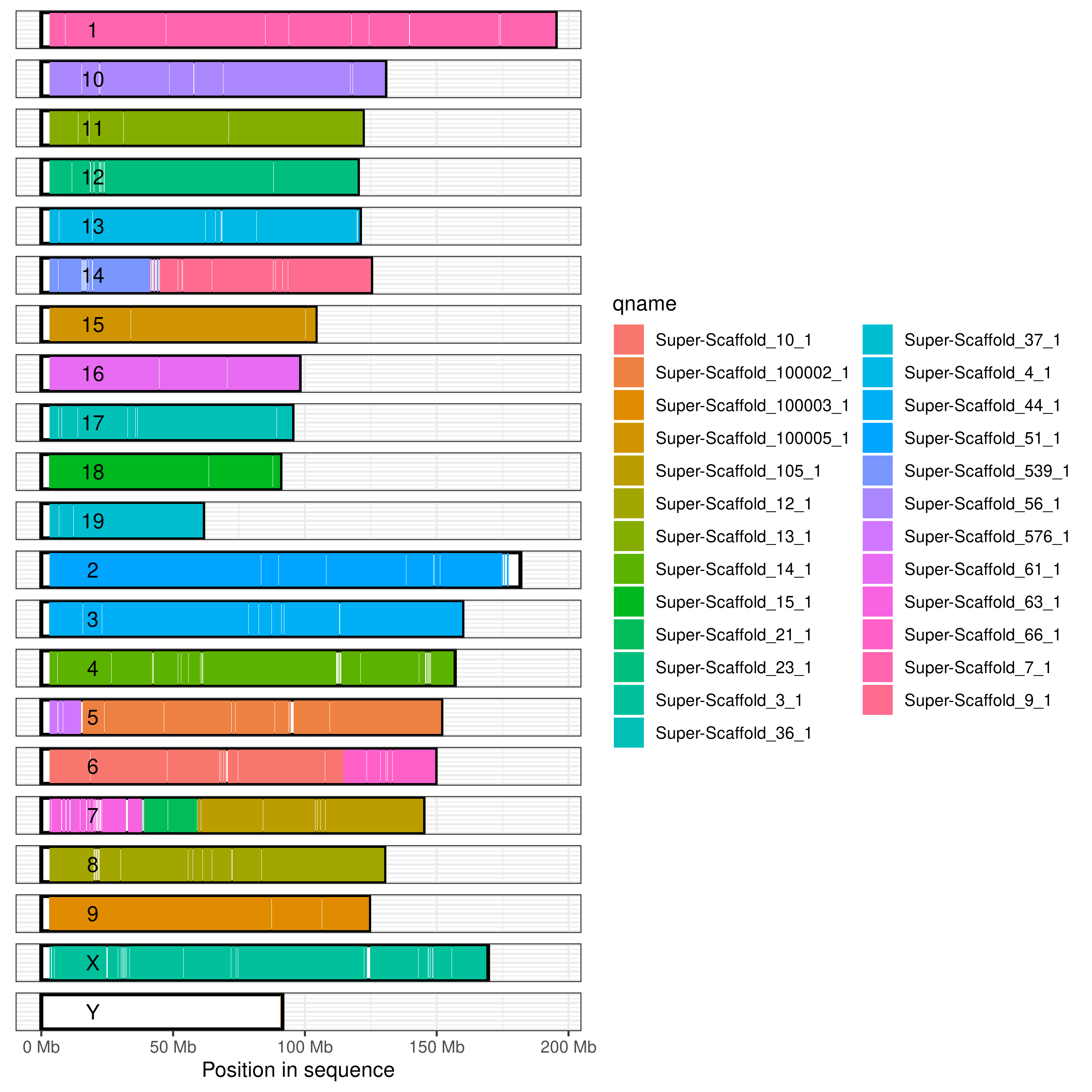

Supplement: jkad188_Supplementary_Data [file jkad188_supplementary_data.zip › Figure_S3_G3-2023-404267.png]

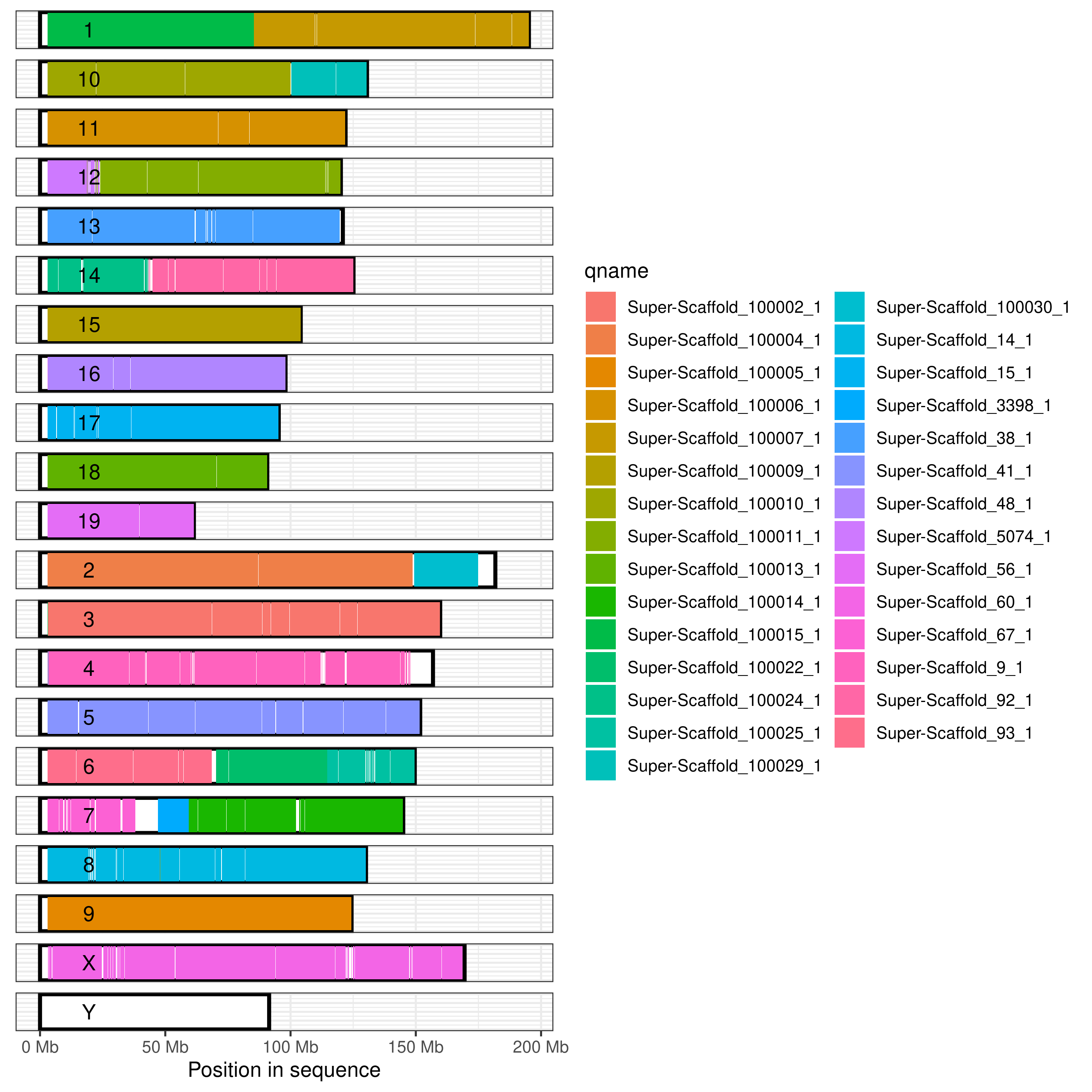

Supplement: jkad188_Supplementary_Data [file jkad188_supplementary_data.zip › Figure_S4_G3-2023-404267.png]
